# ABO non-O type as a risk factor for thrombosis in patients with pancreatic cancer

**DOI:** 10.1002/cam4.513

**Published:** 2015-08-15

**Authors:** Donghui Li, Mayurika N Pise, Michael J Overman, Chang Liu, Hongwei Tang, Saroj Vadhan-Raj, James L Abbruzzese

**Affiliations:** 1 Department of Gastrointestinal Medical Oncology, The University of Texas MD Anderson Cancer CenterHouston, Texas; 2 Department of Sarcoma Medical Oncology, The University of Texas MD Anderson Cancer CenterHouston, Texas

**Keywords:** ABO blood type, pancreatic cancer, risk factors, thrombosis

## Abstract

ABO blood type has previously been identified as a risk factor for thrombosis and pancreatic cancer (PC). The aim of the study is to demonstrate the associations between ABO blood type and other clinical factors with the risk of thromboembolism (TE) in patients with PC. We conducted a retrospective study in 670 patients with pathologically confirmed pancreatic adenocarcinoma at the University of Texas MD Anderson Cancer Center. Clinical information was retrieved from medical records. ABO blood type was determined serologically and/or genetically. Logistic regression models, Kaplan–Meier plot, log-rank test, and Cox proportional hazard regression models were employed in data analysis. The incidence of TE was 35.2% in 670 patients who did not have TE prior to cancer diagnosis. Pulmonary embolism (PE) and deep vein thrombosis (DVT) consisted 44.1% of the TE events. Non-O blood type, pancreatic body/tail tumors, previous use of antithrombotic medication, and obesity (body mass index >30 kg/m^2^) were significant predictors for TE in general. Blood type A and AB, low hemoglobin level (≤10 g/dL), obesity, metastatic tumor, and pancreatic body/tail tumors were significant predictors for PE and DVT. Patients with metastatic tumor or pancreatic body/tail tumors had a much higher frequency of early TE events (≤3 months after cancer diagnosis); and early TE occurrence was a significant independent predictor for increased risk of death. These observations suggest that ABO non-O blood type is an independent predictor for TE in PC. A better understanding of the risk factors for TE in PC may help to identify patients who are most likely to benefit from prophylactic anticoagulation therapy.

## Introduction

Pancreatic cancer (PC) has the highest incidence of venous thromboembolism (VTE) during the first year of the cancer diagnosis among all common human malignancies 1. Cancer patients with TE have a higher risk of recurrence and a poor prognosis 2. Therefore, better understanding of the risk factors associated with development of TE and the effect of TE on survival may help identify the high-risk subgroup of patients for primary thromboprophylaxis. Known risk factors for cancer-associated VTE include primary site of the cancer, presence of metastatic disease, and use of antineoplastic therapy 3,4, and suggestive factors include body mass index (BMI), leukocyte and platelet counts, and level of tissue factor 5. Currently it is not clear what the most important risk factors are for TE in PC and whether the reported risk model can be validated in PC patients.

The ABO gene variants have recently identified as a susceptibility factor for PC 6. This finding was supported by serological evidence that individuals with a non-O blood group had increased risk of PC 7. However, the potential mechanisms by which ABO blood types may influence the risk of PC are unknown. The ABO gene codes for several glycosyltransferases that add sugar residues to the H(O) antigen. In addition to blood cells, the H(O) antigens also reside on the surface of von Willebrand factor (vWF), a carrier protein for coagulation factor VIII (FVIII). Individuals with the ABO A1 and B alleles were found to have a lower rate of vWF clearance, thus higher plasma levels of vWF and FVIII, and higher risk for thrombosis 8. A recent meta-analysis of literature has concluded that non-O blood type was the most common genetic risk factor for VTE among noncancer patients 9. The purpose of the current study is to test the hypothesis that non-O blood type may affect the risk of VTE in patients with PC.

## Material and Methods

Patients involved in the current study were drawn from a previously conducted case–control study of PC at MD Anderson Cancer Center 10 and the study was approved by the Institutional Review Board of MD Anderson. Eligible patients were diagnosed with a pathologically confirmed pancreatic ductal adenocarcinoma and had no prior history of TE. A written informed consent for personal interview, medical record review, and a blood sample collection was obtained from each patient. Epidemiological information on risk factors for PC was collected by personal interview at recruitment.

Clinical information was collected from medical record review, including date of cancer diagnosis, tumor stage (resected/resectable, locally advanced, and metastatic), tumor size (by endoscopic ultrasound or radiographic measurement), tumor site (pancreas head, neck, body, tail, and multisite), tumor grade (well, moderate, or poorly differentiated), tumor resection (yes or no), serum carbohydrate antigen (CA) 19-9 level, performance status at diagnosis, and date of last follow-up or date of death. Any atherosclerosis mentioned in the radiological reports was noted. All radiological reports were derived from imaging analyses conducted at MD Anderson according to the standard clinical protocols. Use of antithrombotic agents like antiplatelet drugs (including aspirin, clopidogrel, dipyridamole, cilostazol, ticlopidine), coumadin and heparin (low molecular weight and unfractionated), was recorded at the initial clinical evaluation of the cancer. Laboratory measurements on complete blood count (CBC), hemoglobin (Hb), and platelet at the time of initial clinical evaluation were collected. Fasting blood glucose levels measured at the time of diagnosis or during the entire cancer course were collected. Initial fasting blood glucose levels and the average of the two highest consecutive readings were used in the analysis.

Information on TE events occurring after cancer diagnosis, the type of anticoagulant used, treatment received 3 months prior to any TE events was collected. TE events were identified based on radiological evidence. These events included deep vein thrombosis (DVT), pulmonary embolism (PE), and others such as portal vein thrombosis and splenic vein thrombosis. The thrombosis locations were classified as PE, DVT in the upper and/or lower limbs, and other sites including thrombosis in arteries and visceral veins.

ABO blood type was determined from medical records for 44% of the patients and was determined by genotyping in the remaining 56% patients using Taqman assay and DNA samples extracted from peripheral lymphocytes. Two single nucleotide polymorphism (SNPs), that is, rs505922 and rs8176746, of the ABO gene were analyzed and the ABO genotypes were inferred as described previously 11.

Univariate logistic regression analysis was performed to identify potential risk factors for TE. Overall survival was estimated from the date of pathological diagnosis to the date of death or date of last follow-up. The maximum time of follow-up was 60 months. All living patients were censored at the time of last follow-up or at the completion of 5-year follow-up. Kaplan–Meier analysis, log-rank test, and Cox proportion hazard regression models were employed in the survival analysis. A forward stepwise approach was adopted for the multivariable Cox regression models. *P* < 0.05 was considered as statistically significant.

## Results

The study population consisted of 670 patients with a median age of 62 years (range 31–87). Of which, 57.9% were men and 88.7% were non-Hispanic whites. There were 202 (30.1%) patients with resected or resectable tumor, 182 (27.2%) with locally advanced, and 286 (42.7%) with metastatic tumor.

TE events were observed in 236/670 (35.2%) patients including 104 (15.5%) PE/DVT. Forty-eight patients had PE, 43 had DVT, and 13 with both (Table1). The majority of other TE events (non-PE and non-DVT) involved portal vein, splenic vein, and superior mesenteric vein. Forty-seven TE events were arterial, 157 were venous, and 32 involving both. Most of the patients (194) had single TE episodes, 39 had two, and three patients had three episodes. The median time from pathological diagnosis of PC to radiological diagnosis of thrombosis was just 4 (range 0–111) months and the median survival time after thrombosis was 6 (range 0–146) months.

**Table 1:** Frequency of different types of TE events

TE locations	*N* (%)
PE + DVT	104 (44.1)
Pulmonary artery embolism only	29 (12.3)
DVT only	21 (8.9)
PE and DVT	13 (5.5)
PE and other veins	19 (8.1)
DVT and other sites	22 (9.3)
Other TE events	
Aorta and other arteries	18 (7.6)
Other vein sites only	114 (48.3)
Portal vein	44 (18.6)
Splenic vein	22 (9.3)
SMV	12 (5.1)
Combined or other veins	36 (15.3)
All events	236 (100)

PE, pulmonary embolism; DVT, deep vein thrombosis; TE, thromboembolism; SMV, superior mesenteric vein.

One-hundred and thirty-nine patients had used some form of antithrombotic treatment, including 98 aspirin, 17 coumadin, 18 clopidogrel, and 6 heparin. In all, 67/139 (48%) of the patients had a history of cardiac artery disease (CAD), cerebral vascular disease (CVD), or peripheral vascular disease (PVD) compared to 42/531 (7.9%) of the patients who did not use any antithrombotic treatment.

A higher risk of TE was associated with tumor stage, tumor size, tumor site, fasting blood glucose, obesity (BMI >30 kg/m^2^), and history of using antithrombotic treatment (Table2). Hb level <10 g/dL was nonsignificantly associated with the risk of TE (*P* = 0.085). The distribution of demographics, history of diabetes, hypertension, CAD, CVD, and PVD, biliary obstruction, performance status, and cancer treatment, presence of atherosclerosis, and levels of CA19-9, CBC, and platelet were comparable between the TE and non-TE group (data are not shown for some variables). Comparing the PE/DVT group with the non-TE group, a statistically significant difference was observed for metastatic tumor, treatment with chemotherapy, tumor size >5 cm, pancreatic body/tail tumors, high blood glucose level, and obesity. A borderline significant difference was detected for Hb level <10 g/dL and previous use of antithrombotic medications.

**Table 2:** Comparison of TE and non-TE groups by univariate analysis

Variable	Non-TE*N* (%)	TE*N* (%)	OR (95% CI)	*P*	PE + DVT*N* (%)	OR (95% CI)	*P*
Age							
≤60	193 (44.5)	98 (41.5)	1.00		45 (43.3)	1.0	
>60	241 (55.5)	138 (58.5)	1.12 (0.82–1.54)	0.463	59 (56.7)	1.05 (0.68–1.62)	0.825
Sex							
Male	249 (57.4)	139 (58.9)	1.00		65 (62.5)	1.0	
Female	185 (42.6)	97 (41.1)	0.94 (0.68–1.30)	0.703	39 (37.5)	0.81 (0.52–1.25)	0.341
Race							
White (non-Hispanic)	389 (89.6)	205 (86.9)	1.00		88 (84.6)	1.0	
Others	45 (10.4)	31 (13.1)	1.31 (0.80–2.13)	0.282	16 (15.4)	1.57 (0.85–2.91)	0.150
Stage at diagnosis							
Resected	139 (32.0)	63 (26.7)	1.00		24 (23.1)	1.0	
Locally advanced	123 (28.3)	59 (25.0)	1.06 (0.69	0.796	21 (20.2)	0.99 (0.53	0.972
Metastatic	172 (39.6)	114 (48.3)	1.46 (1.00	0.050	59 (56.7)	1.99 (1.18	0.010
Treatment							
None	55 (12.7)	25 (10.6)	1.00		6 (5.8)	1.0	
Chemotherapy	147 (33.9)	103 (43.6)	1.54 (0.90	0.113	61 (58.7)	3.80 (1.56	0.003
Chemoradiation	232 (53.5)	108 (45.8)	1.02 (0.61	0.929	37 (35.6)	1.46 (0.59	0.414
Tumor size (cm)^1^							
≤2	64 (15.3)	19 (8.5)	1.00		8 (8.1)	1.0	
>2–5	240 (57.6)	126 (56.5)	1.77 (1.02	0.044	51 (51.5)	1.70 (0.77	0.191
>5	113 (27.1)	78 (35.0)	2.33 (1.29	0.005	40 (40.4)	2.83 (1.25	0.013
Tumor site							
Head	299 (68.9)	132 (55.9)	1.00		58 (55.8)	1.0	
Other sites	135 (31.1)	104 (44.1)	1.75 (1.26–2.42)	0.001	46 (44.2)	1.75 (1.13–2.72)	0.012
CA 19-9 levels (U/mL)^1^							
≤47	76 (17.6)	44 (18.6)	1.00		20 (19.2)	1.0	
48–500	164 (37.9)	79 (33.5)	0.83 (0.53–1.32)	0.432	33 (31.7)	0.77 (0.41–1.41)	0.395
>500	193 (44.6)	113 (47.9)	1.01 (0.65–1.57)	0.960	51 (49.0)	1.00 (0.56–1.80)	0.989
Diabetes							
No	318 (73.3.)	175 (74.2)	1.00		76 (73.1)	1.0	
Yes	116 (26.7)	61 (25.8)	0.96 (0.67–1.37)	0.805	28 (26.9)	1.01 (0.62–1.64)	0.968
Blood glucose (mg/dL)^1^							
≤200	287 (66.3)	130 (55.1)	1.00		57 (54.8)	1.0	
>200	146 (33.7)	106 (44.9)	1.64 (1.19–2.26)	0.002	47 (45.2)	1.62 (1.05–2.50)	0.029
BMI (kg/m^2^)^1^							
≤30	300 (69.8)	146 (62.4)	1.00		53 (51.5)	1.0	
>30	130 (30.2)	88 (37.6)	1.39 (1.00–1.95)	0.054	50 (48.5)	2.18 (1.41–3.37)	<0.001
Hb (g/dL)							
>10	419 (96.5)	220 (93.6)	1.00		96 (92.3)	1.0	
≤10	15 (3.5)	15 (6.4)	1.91 (0.91–3.97)	0.085	8 (7.7)	2.32 (0.96–5.65)	0.062
CBC (K/*μ*L)							
≤11	400 (92.2)	214 (91.1)	1.0		92 (88.5)	1.0	
>11	34 (7.8)	21 (8.9)	1.15 (0.65–2.04)	0.621	12 (11.5)	1.54 (0.77–3.08)	0.228
Platelet (K/*μ*L)^1^							
≤350	360 (83.1)	190 (81.2)	1.0		86 (82.7)	1.0	
>350	73 (16.9)	44 (18.8)	1.18 (0.79–1.76)	0.413	18 (17.3)	1.03 (0.59–1.82)	0.913
Atherosclerosis							
No	319 (73.5)	165 (69.9)	1.00		77 (74.0)	1.0	
Yes	115 (26.5)	71 (30.1)	1.19 (0.84–1.70)	0.322	27 (26.0)	0.97 (0.60–1.58)	0.973
Antithrombotic treatment							
No	356 (82.0)	175 (74.2)	1.00		76 (73.1)	1.0	
Yes	78 (18.0)	61 (25.8)	1.59 (1.09–2.33)	0.017	28 (26.9)	1.52 (0.92–2.53)	0.105

Non-TE, no thromboembolism; TE, thromboembolism; OR, odds ratio; PE, pulmonary embolism; DVT, deep vein thrombosis; CA, carbohydrate antigen; BMI, body mass index; Hb, hemoglobin; CBC, complete blood count.

1 Information on clinical parameters was missing from some patients so the numbers do not add up to the total.

The ABO blood type distribution was 37.5% O, 49.0% A, 10.7% B, and 2.8% AB in this study (Table3). Type O consisted 41.0% of the nonthrombotic group and 30.9% of the thrombotic group. Using type O as the reference group, type A, AB, combined A and AB, and the non-O type were all associated with a significantly increased risk of TE in univariable models. PE/DVT patients had a significantly higher frequency of blood type A and AB (64.9%) than the non-TE patients (48.2%). Type A and AB was associated with a twofold increased risk of PE/DVT.

**Table 3:** ABO blood type and risk of thrombosis in patients with pancreatic cancer

Blood type	All patients*N* (%)	Non-TE*N* (%)	TE*N* (%)	OR (95% CI)^1^	*P*	PE + DVT	OR (95% CI)^1^	*P*
O	251 (37.5)	178 (41.0)	73 (30.9)	1.0		33 (31.7)	1.0	
A	328 (49.0)	200 (46.1)	128 (54.2)	1.56 (1.10–2.22)	**0.013**	56 (53.8)	1.51 (0.94–2.43)	0.089
B	72 (10.7)	47 (10.8)	25 (10.6)	1.30 (0.74–2.26)	0.360	10 (9.6)	1.15 (0.53–2.50)	0.728
AB	19 (2.8)	9 (2.1)	10 (4.2)	2.71 (1.06–6.94)	**0.038**	5 (4.8)	3.00 (0.94–9.51)	0.062
A + AB	347 (51.8)	209 (48.2)	138 (58.4)	1.61 (1.14–2.28)	**0.007**	61 (64.9)	1.99 (1.25–3.16)	**0.004**
Non-O	419 (62.5)	256 (59.0)	163 (69.1)	1.55 (1.11–2.17)	**0.010**	71 (68.3)	1.50 (0.95–2.36)	0.083

Non-TE, no thromboembolism; TE, thromboembolism; OR, odds ratio; PE, pulmonary embolism; DVT, deep vein thrombosis.

1 OR (95% CI) from univariable logistic regression analysis.

In multivariable logistic regression analysis, tumor site, use of antithrombotic medication, non-O blood type, and BMI remained as significant predictors for risk of TE (Table4). The model predicted that the probability of developing TE is 63.9% for patients with all four at-risk factors. Overall, the model was able to correctly classify 93% of the non-TE cases (specificity) and 17% of the TE cases (sensitivity). The positive predictive value (PPV) is 56.3% and negative predictive value (NPV) is 67.3%. When the analysis was restricted to PE and DVT only, Hb ≤10 g/dL, obesity, metastatic tumor, and tumor site as well as blood type A and AB were identified as significant independent risk predictors (Table4). Patients with all five at-risk factors have a probability of 59.8% developing PE/DVT. The model had a specificity of 100%, sensitivity of 2.2%, PPV 100%, and NPV 82.5%.

**Table 4:** Risk factors for TE in pancreatic cancer

Variable	*β*	OR (95% CI)	*P* value
All TE events			
Tumor site (pancreas head vs. body/tail)	0.610	1.84 (1.32–2.57)	<0.001
Blood type (non-O vs. O type)	0.496	1.64 (1.16–2.32)	0.005
Antithrombotic treatment (yes vs. no)	0.468	1.60 (1.08–2.36)	0.019
BMI (≤30 vs. >30 kg/m^2^)	0.386	1.47 (1.04–2.07)	0.028
DVT and PE only			
Hemoglobin (>10 vs. ≤10 g/dL)	1.157	3.18 (1.14–8.85)	0.027
BMI (≤30 vs. >30 kg/m^2^)	0.898	2.45 (1.52–3.97)	<0.001
Stage (nonmetastatic vs. metastatic)	0.599	1.82 (1.11–2.98)	0.017
Tumor site (pancreas head vs. body/tail)	0.560	1.75 (1.06–2.90)	0.030
Blood type (O vs. A/AB)	0.555	1.74 (1.07–2.84)	0.027

TE, thromboembolism; OR, odds ratio; BMI, body mass index; DVT, deep vein thrombosis; PE, pulmonary embolism.

**Figure 1:**
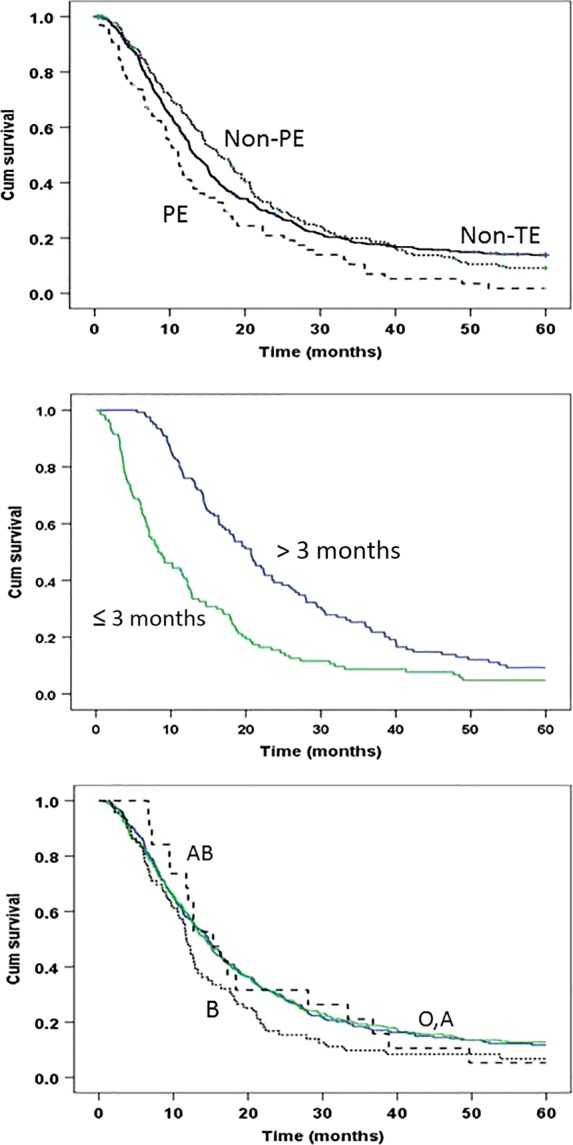
Kaplan–Meier plot of overall survival time by thrombotic status (upper panel), lagging time between cancer diagnosis to thrombosis (middle panel), and ABO blood types (lower panel). Non-TE, nonthrombotic; PE, pulmonary thromboembolism; non-PE, thromboembolism of nonpulmonary sites. Information on median survival time, hazard ratios (95% CI), and *P* values are presented in Table5.

Thrombosis in general was not significantly associated with the overall survival time or the risk of death in this patient population (Table5). However, univariable analysis showed that patients with PE had a significantly shorter survival and increased risk of death than those with other types of TE or no TE (Fig.1 and Table5), but the significance disappeared after adjusting for tumor stage and ABO blood type (HR = 1.24, 95% CI = 0.94–1.53, *P* = 0.135). Patients with TE occurred ≤3 months after the cancer diagnosis had a much poorer survival than those with the events occurred >3 months after the cancer diagnosis (Fig.1). The time to TE occurrence, however, was significantly influenced by the tumor site and tumor stage. Patients with early TE events had a much higher frequency of metastatic tumor (63.8%) and tumors of pancreas body and/or tail (55.2%) than those with a late TE event (33.3%). Nevertheless, an early TE event remained as a significant predictor for risk of death after adjusting for tumor stage and tumor site (HR = 1.78, 95% CI = 1.35–2.35, *P* < 0.001).

**Table 5:** Impact of thrombosis and ABO blood type on overall survival

Variable	MST (95% CI) (months)	*P* (log-rank)	Hazard ratio (95% CI)^1^	*P* value
TE				
No	13.0 (11.6–14.5)		1.00	
Yes	14.3 (12.2–16.5)	0.394	1.08 (0.91–1.27)	0.395
Pulmonary	10.2 (8.0–12.4)	0.011	1.49 (1.13–1.97)	0.004
PE + DVT	12.3 (10.1–14.4)	0.014	1.34 (1.07–1.67)	0.010
Others	17.1 (13.6–20.6)	0.444	0.92 (0.75–1.14)	0.922
Time of TE^2^				
>3 months	20.3 (17.3–23.3)		1.0	
≤3 months	8.5 (6.3–10.7)	<0.001	2.12 (1.62–2.77)	<0.001
ABO blood type				
O	14.3 (12.4–16.3)		1.00	
A	14.2 (12.6–15.8)	0.848	0.98 (0.82–1.17)	0.842
B	11.6 (10.2–13.0)	0.076	1.29 (0.98–1.70)	0.071
AB	12.9 (9.2–16.7)	0.936	1.02 (0.63–1.65)	0.934

MST, median survival time; PE, pulmonary embolism; DVT, deep vein thrombosis; TE, thromboembolism.

1 Hazard ratio (95% CI) from univariable Cox regression analysis.

2 Time from cancer diagnosis to TE occurrence.

Non-O blood types were not associated with overall survival (Table4). Even though blood type B was not associated with the risk of TE, it showed a reduced overall survival and increased risk of death compared with other blood types (Table5). Nevertheless, these differences were not statistically significant after adjusting for known clinical predictors such as tumor stage, performance status, and serum CA19-9 level (data not shown).

## Discussion

In this study, we found that the non-O blood types compared to type O, especially type A and AB, was associated with significantly increased risk for thrombosis in PC patients. Other risk predictors for TE in this patient population included tumor of the pancreatic body and tail, obesity, metastatic tumors, Hb <10 g/dL, and use of antithrombotic medications. These findings will be useful in building a risk prediction model for identifying patients with high-risk of thrombosis in PC.

Previous studies have conclusively linked the ABO locus to risk of PC 6, VTE 12, and myocardial infarction in the presence of coronary atherosclerosis 13. The current study for the first time demonstrated that ABO blood type was an independent predictor for risk of TE among PC patients. ABO blood group is a major determinant of coagulant factor VIII and vWF plasma levels 8,14. Type O individuals have ∼25% lower plasma levels of these glycoproteins than type A individuals, which may account for the reduced risk of TE. Furthermore, ABO locus has been associated with circulating levels of soluble intercellular adhesion molecule-1, soluble P-selectin, soluble E-selectin, and to other markers known to participate in inflammatory processes 15,16. Although the underlying mechanisms that linking ABO locus to these glycoproteins are not fully understood at this point, it is plausible that the level of these proteins could be directly related to the development of thrombosis among patients with PC.

A previous study has reported an increased risk of TE in association with metastatic tumor and tumor of the pancreatic body and tail 17. We made similar observations in the current study. The deleterious effect could be explained by the finding that cancers of the body or tail are often characterized by increasing transperitoneal and hematogenous spread to a greater extent than are those of the head 18.

Prior use of antithrombotic medications was associated with increased risk of TE. Patients who used antithrombotic medication had a much higher frequency of cardiovascular comorbidities than those who did not use it. Although none of these diseases was identified as a risk factor for TE in this patient population, these conditions share some common risk factors with TE, such as obesity and non-O blood type. Use of antithrombotic medications is perhaps a reflection of the vascular diseases and the underlying risk factors that actually contributed to the development of TE.

Consistent with previous findings, obesity (BMI >30 kg/m^2^) was significantly associated with increased risk of TE in general (odds ratio [OR] = 1.47, 95% CI = 1.04–2.08, *P* = 0.028) and with PE and DVT in particular (OR = 2.49, 95% CI = 1.55–4.02, *P* < 0.001) in this study. Plausible mechanisms linking obesity to risk of TE include the physical effects of body fat limiting venous return and a proinflammatory, prothrombotic, and hypofibrinolytic milieu 19.

Diabetes can be either a risk factor or a consequence of the PC 20. Patients with PC often have concurrent diabetes or impaired glucose tolerance. Diabetes has been suggested to have a negative impact on the clinical outcome of PC 21,22. In this study, we did not detect an association between TE and history of diabetes or initial blood glucose level. However, patients with a blood glucose level >200 mg/dL documented at any time during the cancer course had a twofold increased risk of TE. The high level of glucose was not associated with tumor stage or ABO blood type (data not shown). It is known that noncancer patients with diabetes mellitus are at increased risk of thrombosis. This is thought to be due to the presence of insulin resistance and an increased inflammatory state which directly affects platelet function, coagulation factors, and clot structure 23.

The high incidence of TE in PC poses considerable challenges to the physician. Available literature suggests that TEs are generally poor prognostic markers due to their allied complications and the associated aggressive tumor histology 24. A previous study in 1915 PC patients showed that developing an early TE significantly increased the risk of death 25. The current study did not observe a significant impact of TE in general on overall survival as compared to the non-TE group. Nevertheless, when TE occurred shortly (≤3 months) after the cancer diagnosis such patients had a much poorer survival and higher risk of death (HR = 2.12, 95% CI = 1.62–2.77, *P* < 0.001) than those patients when the TE events occurred >3 months after the cancer diagnosis.

Overall, we have identified ABO blood type, along with tumor stage, tumor site, BMI, Hb level, and previous use of anticoagulation medication as predictors for risk of TE in PC. The risk prediction models built based on these factors showed a high specificity but rather low sensitivity, suggesting that the model has a low false-positive rate so the small number of high-risk patients identified by the model should truly benefit from prophylactic treatment. In addition, biomarkers such as tissue factor or tissue-factor-positive microparticles, need to be considered to improve the predictive power 26,27. The strength of the study includes a large sample size and detailed clinical information collected from a single hospital. The limitations of the study include its retrospective design, heterogeneity of the patient population, and lack of sufficient details on time variables, for example, time between chemotherapy or radiotherapy and TE events. Identification of predictive risk factors is a strategy for appropriate and timely initiation of anticoagulation treatment 5,28. Further research is required to identify subgroups associated with TE who would benefit with anticoagulation.
